# Retrieval-augmented Chinese text-to-SQL generation for conversational bibliographic search

**DOI:** 10.1371/journal.pone.0334965

**Published:** 2025-10-27

**Authors:** Zhenyu Wang, Mark Xuefang Zhu, Guo Li, Shanshan Kong

**Affiliations:** School of Information Management, Nanjing University, Nanjing, Jiangsu, China; Philadelphia University, JORDAN

## Abstract

To overcome the limitations of current bibliographic search systems, such as low semantic precision and inadequate handling of complex queries, this study introduces a novel conversational search framework for the Chinese bibliographic domain. Our approach makes several contributions. We first developed BibSQL, the first Chinese Text-to-SQL dataset for bibliographic metadata. Using this dataset, we built a two-stage conversational system that combines semantic retrieval of relevant question-SQL pairs with in-context SQL generation by large language models (LLMs). To enhance retrieval, we designed SoftSimMatch, a supervised similarity learning model that improves semantic alignment. We further refined SQL generation using a Program-of-Thoughts (PoT) prompting strategy, which guides the LLM to produce more accurate output by first creating Python pseudocode. Experimental results demonstrate the framework’s effectiveness. Retrieval-augmented generation (RAG) significantly boosts performance, achieving up to 96.6% execution accuracy. Our SoftSimMatch-enhanced RAG approach surpasses zero-shot prompting and random example selection in both semantic alignment and SQL accuracy. Ablation studies confirm that the PoT strategy and self-correction mechanism are particularly beneficial under low-resource conditions, increasing one model’s exact matching accuracy from 74.8% to 82.9%. While acknowledging limitations such as potential logic errors in complex queries and reliance on domain-specific knowledge, the proposed framework shows strong generalizability and practical applicability. By uniquely integrating semantic similarity learning, RAG, and PoT prompting, this work establishes a scalable foundation for future intelligent bibliographic retrieval systems and domain-specific Text-to-SQL applications.

## Introduction

Bibliographic search, as a critical component of library reference services, directly impacts user experience and resource utilization efficiency through its level of intelligence. The motivation for this work stems from the significant shortcomings of current bibliographic search services. Firstly, the degree of intelligence remains limited. Traditional systems primarily rely on rule-based methods, requiring users to translate their information needs into pre-defined combinations of system rules. While this approach can handle simple queries, it suffers from high maintenance costs and poor scalability of the rule base. Secondly, the service model is relatively passive, lacking in-depth understanding of users’ search intentions. When user queries go beyond the scope of the bibliographic database, the system fails to provide effective support, severely affecting the service experience. In contrast, conversational question-answering systems based on natural language processing (NLP) technologies can gradually clarify user needs through multi-turn interactions. This model has already demonstrated significant advantages in fields such as e-commerce customer service [1] and medical consultation [2].

Large language models (LLMs) have advanced Text-to-SQL tasks with strong semantic parsing capabilities [3]. Through in-context learning and fine-tuning, LLMs significantly boost performance [4]. In-context learning, in particular, enables effective zero-shot or few-shot adaptation, making it well-suited for data-scarce conversational bibliographic retrieval [5]. Studies show few-shot prompts outperform zero-shot ones in enhancing LLM performance [6], prompting the development of retrieval-augmented frameworks for better demonstration selection. Despite success on English benchmarks like BIRD and Spider, challenges remain in applying these methods to Chinese bibliographic scenarios. User queries in bibliographic search often share structural patterns or express similar meanings with varied wording. General-purpose embedding models struggle with subtle semantic differences in domain-specific contexts, reducing retrieval accuracy. Moreover, some queries require recursive search or multi-table joins, which can lead to syntax errors or incomplete logic in LLM-generated SQL. While such logic can be easier to express in Python pseudocode, this highlights the need for new generation strategies or intermediate representations to lower complexity. Finally, there is still a lack of high-quality, annotated Chinese datasets tailored for Text-to-SQL in the bibliographic domain.

To address these challenges, this paper introduces a novel framework for conversational bibliographic search with several key contributions:

1. **Creation of the BibSQL Dataset:** We construct and release BibSQL, the first publicly available Chinese Text-to-SQL dataset specifically designed for the bibliographic domain, providing a valuable resource for future research.
2. **Development of SoftSimMatch:** We propose a supervised semantic similarity learning framework, SoftSimMatch, which is fine-tuned on domain-specific data to significantly improve the accuracy of retrieving relevant examples for in-context learning.
3. **Novel Application of PoT Prompting:** We are the first to apply and evaluate a Program-of-Thoughts (PoT) [7] prompting strategy in the bibliographic search domain, demonstrating its effectiveness in improving SQL generation accuracy, especially in low-resource settings.
4. **Comprehensive Framework and Evaluation:** We build and comprehensively evaluate an end-to-end system that integrates these components, demonstrating its state-of-the-art performance and practical applicability on a variety of modern open-source LLMs.

Building on BibSQL, we develop a conversational retrieval system that follows a two-stage workflow: first retrieving relevant question-SQL pairs via semantic similarity, then guiding LLMs to generate executable SQL using in-context learning templates. We apply the PoT technique to help LLMs produce optimized SQL queries with support for custom function syntax.

## Related work

### Conversational search system

With the continuous advancement of natural language processing and information retrieval technologies, conversational search systems have attracted widespread attention as a new retrieval paradigm [8–10]. These systems aim to iteratively clarify user needs and enhance the search experience through multi-turn interactions. Unlike traditional search engines (e.g., Google, Bing) that rely on keyword matching, conversational search systems focus on understanding user intent, maintaining dialogue context, and dynamically adjusting query strategies during the interaction process. This allows users to express their information needs more naturally, making conversational search particularly suitable for scenarios involving complex information needs and unclear search goals [11,12].

As the demand for specialized retrieval in various vertical domains continues to grow, conversational search technologies are evolving towards domain-specific applications [13]. In bibliographic search tasks, the data structures are often complex, and the retrieval needs are highly specialized. Users frequently need to iteratively adjust filtering criteria to obtain high-quality results. Against this backdrop, conversational bibliographic search (CBS) has emerged as a concrete application of conversational search systems in the domain of academic bibliographic retrieval. CBS aims to iteratively refine queries through multi-turn natural language interactions, thereby improving both the effectiveness of academic resource retrieval and the overall user experience.

Existing CBS systems typically establish connections with databases through slot-filling mechanisms [14]. By identifying user intents and extracting entities from user input, these systems structure retrieval needs into query conditions that databases can process. This approach offers good controllability and interpretability in handling simple queries. However, it reveals significant limitations in complex, multi-turn, and semantically ambiguous academic search scenarios. On one hand, slot definitions heavily rely on domain-specific prior knowledge, making it difficult to cover long-tail demands and complex expressions. On the other hand, the lack of deep semantic alignment between natural language and database schemas often leads to slot-filling errors or query deviations, ultimately affecting retrieval performance [15].

As the scale and structural complexity of academic bibliographic databases continue to grow, relying solely on slot-filling methods is increasingly insufficient to meet users’ expectations for high-quality search results. This has driven researchers to explore more generalized natural language interfaces. Among them, the automatic translation of user dialogue into executable SQL queries (Text-to-SQL generation) has become a key direction for CBS systems to overcome the bottleneck in retrieval accuracy. Kreutz et al. [16] proposed SchenQL, a query language designed for bibliographic metadata, which enables users to perform queries more easily and accurately. With the support of conversational search systems, users should be able to search bibliographic metadata effectively without requiring prior knowledge of query languages.

Driven by LLMs and knowledge-enhanced retrieval technologies, conversational search systems are increasingly integrating query construction methods based on text-to-SQL generation. This approach enhances the system’s ability to understand and express complex queries while maintaining a natural language interaction experience [17,18]. Especially in multi-turn dialogue scenarios, conversational search systems effectively mitigate the limitations of traditional slot-filling methods in terms of flexibility, robustness, and scalability through context-aware query rewriting and semantic matching [19].

As the scale and structural complexity of academic bibliographic databases continue to grow, relying solely on slot-filling methods is increasingly insufficient to meet users’ expectations for high-quality search results. This has driven researchers to explore more generalized natural language interfaces. Among them, the automatic translation of user dialogue into executable SQL queries (Text-to-SQL generation) has become a key direction for CBS systems to overcome the bottleneck in retrieval accuracy.

### Text-to-SQL generation

The Text-to-SQL generation task aims to automatically translate natural language queries into structured SQL statements, serving as a crucial technology for connecting natural language interfaces to databases. Compared to early approaches relying on handcrafted rules and templates, neural network-based methods have significantly improved the model’s ability to handle complex query structures and ambiguous expressions [20]. However, due to the lexical ambiguity, flexible word order, and domain-specific terminology inherent in the Chinese language, directly applying English-oriented Text-to-SQL methods performs poorly in Chinese scenarios [21].

To address these challenges, researchers have proposed large-scale Chinese datasets such as TableQA [22], DuSQL [23], CHASE [24], and SeSQL [25], covering a wide range of scenarios from single-turn to multi-turn question answering and querying. These efforts have significantly contributed to the standardization and evaluation frameworks of Chinese Text-to-SQL tasks [26]. However, existing work primarily focuses on structured table queries and simple database interfaces, and has yet to explore scenarios involving complex metadata, multi-level semantics, and dynamic query expressions in bibliographic retrieval.

In recent years, retrieval-augmented generation (RAG) techniques have been increasingly adopted in Text-to-SQL tasks as an effective means to address challenges related to data scarcity and semantic alignment. By incorporating external knowledge bases (such as Wikidata) or similar query examples, RAG methods significantly enhance the model’s generalization capability across domains, long-tail requirements, and complex expressions [27]. When combined with large-scale pretrained language models, such approaches benefit from both vast linguistic generalization and domain-specific structural knowledge [28]. In the healthcare domain, Ziletti et al. [29] were the first to apply RAG techniques to epidemiological question answering based on electronic health records. By integrating medical coding with the text-to-SQL generation process, they validated the substantial performance improvements achieved by combining GPT-4 Turbo with RAG, offering a novel approach for small-scale data scenarios in vertical domains.

LLMs have recently emerged as a dominant force in text-to-SQL research owing to their ability to capture rich semantic and syntactic patterns from vast corpora. These models support in-context learning through carefully designed prompts that encode schema information, database content, and domain-specific heuristics [30]. In such systems, constructing dynamic prompts that adapt to multi-turn conversational contexts is essential for managing the evolution of user queries during interactive search sessions [31]. Methods such as DART-SQL [32] have achieved significant improvements in table-based question answering and business data query tasks by introducing execution-guided refinement based on database feedback. Abacus-SQL [33] and XRICL [34] enhance model robustness and few-shot transferability through data augmentation and cross-lingual example retrieval, respectively. Xia et al. [35] proposed a consensus-based multi-agent framework, where collaborative optimization between SQL authoring agents and reviewing agents surpasses traditional single-LLM approaches on benchmarks like Spider and Bird. This method notably improves the performance of open-source models such as Llama-3-8B, offering a new paradigm for ensuring the quality of complex query generation.

**Uniqueness of the present work.** While previous studies have advanced Text-to-SQL generation through retrieval augmentation and in-context learning, our work is unique in several respects. First, we address the data scarcity problem in the specialized domain of Chinese bibliographic search by creating and releasing the BibSQL dataset. Second, unlike methods that rely on general-purpose embedding models, we introduce SoftSimMatch, a supervised similarity learning framework tailored to capture the specific semantic nuances of bibliographic queries. Finally, our framework is the first to systematically integrate and evaluate the PoT strategy in this context, demonstrating a practical approach to decomposing complex logic and improving generation accuracy. This combination of a domain-specific dataset, a specialized retrieval model, and an advanced prompting strategy distinguishes our work from existing literature and provides a comprehensive solution for building intelligent conversational search systems in this domain.

## BibSQL dataset creation

Due to the current lack of publicly available Text-to-SQL datasets in the field of bibliographic retrieval, this section will provide a detailed description of our method for constructing question-SQL pairs based on bibliographic data.

### Bibliographic database construction

The bibliographic database constructed in this study is based on the collection data of Nanjing University Library, supplemented with information from online sources such as Douban Books and encyclopedic knowledge graphs. The construction process is as follows: First, a diverse bibliographic dataset containing 100,000 records was obtained from Nanjing University Library. Second, missing metadata in the collection data was supplemented through the Douban platform, e.g., user ratings, thematic tags, and reader reviews. Finally, author background information was extracted from encyclopedic knowledge graphs to enrich author profiles. The database adopts a relational structure design, comprising two primary tables: the book information table and the author information table, which are linked through the author’s name field.

### BibSQL generation

**Fig 1:**
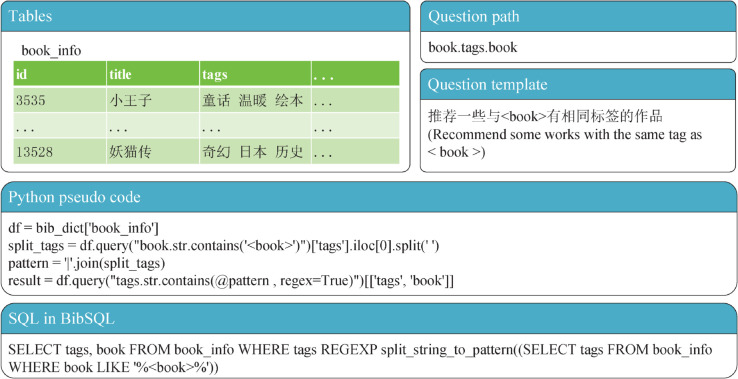
A multi-hop example from the BibSQL dataset.

In the absence of publicly available user query datasets in the domain of bibliographic search, we first constructed a set of basic questions based on the two aforementioned tables, comprising 26 types of single-hop questions and 17 types of multi-hop questions. Each question type corresponds to a specific question path, designed according to the following rules: single-hop questions follow the format of <start node.target node>, while multi-hop questions adopt the format of <start node.intermediate node.target node> (as illustrated by the example of a multi-hop BibSQL question in Fig 1).

To further expand the dataset, we combined pairs of single-hop question paths to generate 76 types of composite question paths. Each question path consists of one SQL query template and multiple natural language question templates. The SQL query templates simplify complex queries through the use of custom function syntax. Thanks to large-scale pretraining, LLMs possess extensive knowledge of programming and databases, enabling them to learn and apply these custom syntax rules through contextual learning.

Given the diversity of path combinations, relying solely on domain experts to manually create natural language questions would be both time-consuming and inefficient. Therefore, we adopted a two-step approach to leverage LLMs for assisting in the generation of question templates:

(1) The questions in BibSQL are manually constructed and categorized into four types based on query complexity: single-hop book metadata search, multi-hop book metadata search, single-hop author metadata search, and multi-hop author metadata search. Some of these queries involve complex operations such as multi-table joins and custom functions. For each type of query path, we designed predefined question templates. Taking the book.author path as an example, we created five different template variations, including expressions like “Who is the author of The Stranger?” and “Who wrote The Stranger?”. Within these templates, specific type placeholders are used to replace concrete condition values (e.g., the book title “The Stranger”). As shown in Table 1, these templates are designed to cover as many common question formulations as possible, thereby enhancing the system’s ability to understand question semantics and improving Text-to-SQL parsing performance.

**Table 1:** Examples of question templates and their corresponding query paths in the BibSQL dataset.

Question path	Question templates
book.summary	What story does <book> tell?
	What does <book> write about?
	Summary of <book>
book.genre.book	What are the works in the same genre as <book>?
	Recommended some works in the same genre as <book>
	Books in the same genre as <book>
book.author.birthplace	What is the birthplace of the author of <book>?
	Do you know where the author of <book> was born?
	Please tell me where the author of <book> is from.

The question templates were originally written in Chinese and translated into English for presentation.

(2) In the question generation stage based on LLMs, we incorporated the manually crafted natural language questions from the first stage into the prompt template. The template is designed to guide the model to generate five different paraphrased variants for each type of question. The manually designed templates serve as demonstration examples, ensuring the normative quality of generated questions while simulating the diverse questioning styles of real users. Through this approach, we achieve both diversity and naturalness in question formulations while maintaining semantic consistency.

### BibSQL statistics

**Fig 2:**
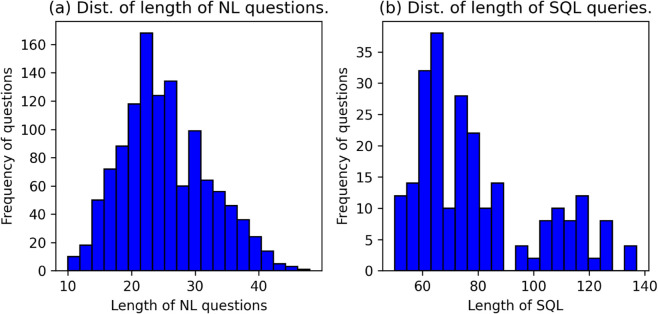
Distribution of questions and queries in the BibSQL dataset. These histograms show the length distributions for (a) natural language questions and (b) SQL queries.

The dataset comprises a total of 119 question types, with each type associated with 10 question templates. Half of these templates are manually written, while the other half are automatically generated by LLMs. Table 2 presents detailed statistical information about the dataset. Fig 2 show the length distributions of natural language questions and SQL queries. To promote transparency and future research, a minimal anonymized version of this dataset has been made publicly available at ScienceDB.

**Table 2:** Detailed statistics of the BibSQL dataset.

Data	Value
# of books	100,000
# of tables	2
# of columns in tables (book, author)	11/8
# of question types	119
# of question-SQL pairs	1190
Average question length (in chars)	25.26
Average SQL length (in chars)	79.87

## Methods

**Fig 3:**
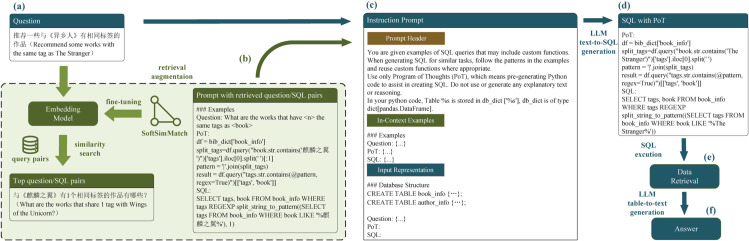
End-to-end workflow of the proposed retrieval-augmented Text-to-SQL framework.

As shown in Fig 3, our approach leverages prompts to LLMs to transform natural language questions into SQL queries. This method overcomes the limitations of traditional text-to-SQL approaches based on exact or string matching, enabling a more comprehensive capture of the semantic complexity inherent in bibliographic search queries. To achieve this, we introduce a critical intermediate step: before generating the SQL query, we guide the LLM using a PoT prompting strategy to first produce Python code (as illustrated by the pseudocode in Fig 1). This intermediate code then serves as the foundation for generating more accurate SQL queries. The resulting queries are executable and directly usable for database information retrieval.

Inspired by the success of RAG in enhancing LLM performance on complex natural language processing tasks, we construct example knowledge base using a subset of the dataset introduced in Section 3. The system retrieves relevant question-SQL pairs from this knowledge base and incorporates them as in-context examples to further refine the SQL generation process. The final SQL queries are executed on a SQLite database with registered custom functions, enabling efficient data retrieval. When needed, users can employ additional LLM prompts to interpret the retrieved results and generate natural language answers.

LLMs have emerged as a powerful paradigm for the Text-to-SQL task, offering effective SQL semantic parsing via in-context learning. Fig 3 illustrates the workflow of our proposed retrieval-augmented Text-to-SQL framework. This section focuses on two core challenges in in-context learning: example selection and example organization.

To begin, we define in-context learning in the context of Text-to-SQL. Given a set of triples $Q=\{(q_{i},s_{i},P_{i})\}$, where *q*_*i*_ denotes a natural language question, *P*_*i*_ is the associated PoT followed by the corresponding SQL query *s*_*i*_, the objective is to maximize the probability that the language model *M* generates the correct SQL query s^*^ for a new target question *q* over a database *D*:

$$\max_{Q^{\prime },\sigma }P_{M}(s^{*}|\sigma (q,D,Q^{\prime })),\;\text{s.t.}|Q^{\prime }|=k\text{ and }Q^{\prime }\subset Q$$ (1)

where σ(·,·,·) denotes the constructed prompt for *q*, which integrates the schema of *D* and *k* selected examples from the set *Q*. The in-context learning process thus hinges on selecting the most informative examples and structuring them effectively within the prompt.

We summarizes several strategies for example selection. The first is random selection, which involves randomly sampling *k* examples from the pool of available candidates. Previous studies have commonly adopted this as a baseline strategy for example selection.The second is question similarity selection, which chooses the *k* examples most similar to the target question. Specifically, a pretrained language model is first used to embed the example questions from the set *Q* and the target question *q*. Then, a predefined distance metric, such as Euclidean distance or cosine similarity, is applied to each example-target pair. Finally, the k-nearest neighbor (KNN) algorithm is used to select the *k* examples from *Q* that best match the target question *q*.

The third strategy is masked question similarity selection, which is designed for Text-to-SQL tasks in bibliographic search. In this method, condition values that are irrelevant to the structure of the transformed SQL query are replaced with generic placeholders across all questions. Similarity is then computed between the masked question embeddings, effectively eliminating the adverse impact of condition values on similarity measurement. It is worth noting that, unlike English, Chinese does not separate words with spaces, making it necessary to perform word segmentation prior to applying masked question similarity selection. However, conventional word segmentation tools often struggle to accurately identify domain-specific Chinese terms.

**SoftSimMatch framework.** To address this challenge, We adopt the SoftSimMatch framework to fine-tune a sentence embedding model on semantically labeled question pairs derived from the BibSQL dataset. This framework integrates both fine-grained label construction and a similarity-based training objective to improve the semantic alignment of sentence embeddings.

**Fig 4:**
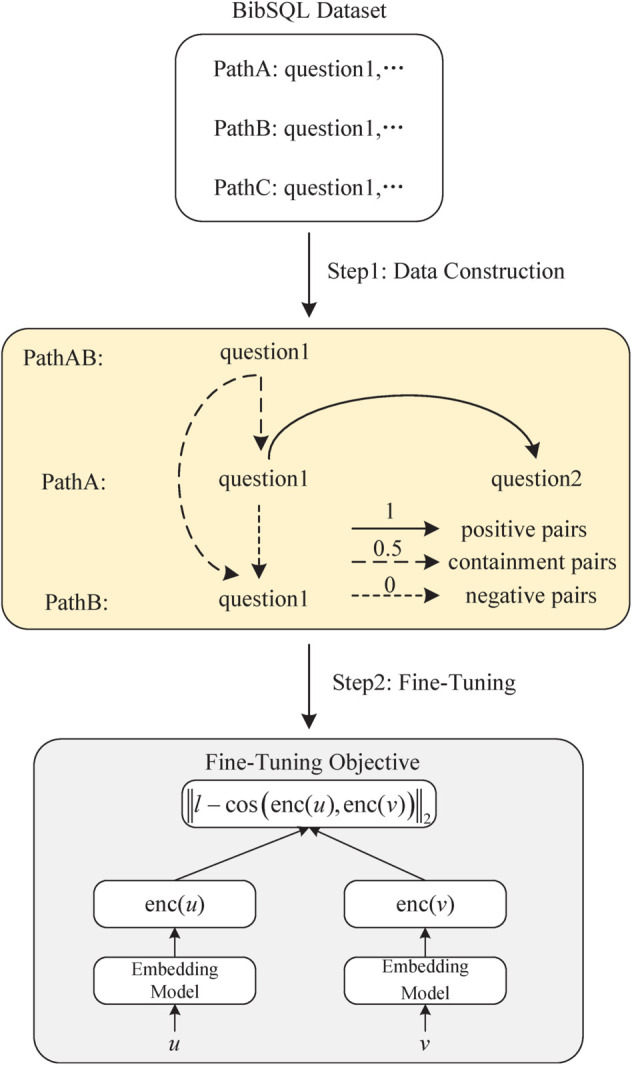
The overall process of the SoftSimMatch framework.

As shown in Fig 4, the SoftSimMatch framework consists of two major steps:

(1) Training data construction. We construct the training data to reflect three levels of semantic relationships between sentence pairs:

- Positive pairs (label = 1): Questions under the same question path are treated as semantically equivalent.
- Negative pairs (label = 0): Using a node-based negative sampling strategy, we identify similar paths that share head/tail nodes and have the same number of nodes. We cross-pair questions from each path to generate semantically distinct question pairs.
- Neutral pairs (label = 0.5): For two related paths A and B, we construct a composite path AB. Questions from A and B are then individually paired with questions from AB to represent semantic containment or entailment.

This labeling scheme supports the learning of more fine-grained and interpretable similarity scores.

(2) Fine-tuning objective. Given a sentence pair (*u*,*v*), we obtain their embeddings using a shared encoder (e.g., SBERT) and compute the cosine similarity. The model is optimized to minimize the squared error between the predicted similarity and the soft label:

$$ℒ={\left\|l-\cos\left(\text{enc}(u),\text{enc}(v)\right)\right\|}_{2}$$ (2)

where *l* represents the similarity label of the sentence pair. enc(*u*) and enc(*v*) represent the embedding vectors of sentence *u* and sentence *v* respectively. $∥\cdot {∥}_{2}$ represents the L2 norm operation.

We first embed both the examples in *Q* and the target question *q* using the text embedding model fine-tuned with SoftSimMatch. Cosine similarity is then computed to evaluate the relevance between each example and the target question. The top-*k* most similar examples are subsequently selected.

Once selected, examples are efficiently formatted to optimize model performance and token usage. To maintain the alignment between questions and their corresponding SQL queries, full question-query pairs are preserved. At the same time, database schemas are omitted from these examples to save tokens.

Each prompt is composed of three parts: a header with a natural language task description that includes instructions for using PoT and custom functions; a set of in-context examples derived from the selected data; and an input representation that presents the target question alongside the full schema of the database *D*, leaving space for the model to generate the corresponding SQL query.

**Self-correction mechanism.** To enhance the robustness of our framework, we introduce a self-correction mechanism for handling query failures or unexpected results. Let $s=\text{LLM}(\sigma (q,D,Q^{\prime }))$ be the initial SQL query generated by the LLM. To ensure this query is executable and to correct it when errors occur, we maintain a conversational context *C*, which is initially set as $C=[\sigma (q,D,Q^{\prime }),s]$. If an execution error occurs or the query returns no results, we append a error description *d* to the context: C←C+d. The LLM is then prompted to generate a corrected SQL query $s^{\prime }$ based on this updated context: $s^{\prime }=\text{LLM}(C)$. The new query $s^{\prime }$ is then concatenated to the context, C←C  +  $s^{\prime }$, and the process is repeated. To manage the context window limit of the LLM, the context *C* is truncated if it exceeds the length limit. This iterative refinement loop continues until a valid query is produced or a maximum of *N* iterations is reached. The complete workflow is detailed in Algorithm 1.

**Algorithm 1** Retrieval-Augmented Text-to-SQL Generation with Self-Correction

**Input:** Question *q*, Database *D*, Example Set *Q*, Max Iterations *N*

**Output:** Final SQL query *s*

1: $Q^{\prime }\leftarrow \text{SelectTopK}(q,Q,k)$ ⊳ Retrieve k similar examples

2: $prompt\leftarrow \text{ConstructPrompt}(q,D,Q^{\prime })$

3: C←[prompt] ⊳ Initialize context

4: n←0

5: s←LLM(C)

6: C←C+s

7: **loop**

8:   is_executable,result←ExecuteSQL(s,D)

9:   **if** is_executable **and** *result* is not empty **then**

10:    **return** *s* ⊳ Success

11:   **end if**

12:   n←n+1

13:   **if** n≥N **then**

14:    **return** *s*

15:   **end if**

16:   d←GenerateErrorDescription(is_executable,result)

17:   C←C+d

18:   s←LLM(C) ⊳ Generate corrected query

19:   C←C+s

20: **end loop**

## Experiments and results

### Experimental setup

#### Dataset description.

The BibSQL dataset, including its question templates and corresponding SQL templates, was used for system evaluation. This dataset comprises 43 basic question types and 76 compound question types. Each question type includes 10 question templates, five manually crafted and five generated by DeepSeek. To ensure that the training and test sets contain no duplicate templates and maintain semantic diversity, the BibSQL dataset is divided into two parts: templates generated by the LLM are used as the test set, while manually created templates form the training set.

After the division of templates, angle brackets and their enclosed value type placeholders were replaced with concrete condition values. This replacement was based on the mappings established between table column names and question paths during the construction of the bibliographic database. From the 100,000 book records and associated author records generated from the bibliographic database, condition values with matching types were randomly selected for template instantiation. One question is generated per template, resulting in a complete dataset of 1,190 questions (595 for training and 595 for testing).

Additionally, to fine-tune the embedding model, an embedding fine-tuning dataset was constructed using the data construction strategy described in Section 4.1 and the BibSQL training set. This dataset contains 5,500 pairs of samples and is randomly split into training and test subsets in an 8:2 ratio. The ratio of positive, negative, and neutral sample pairs is 4:4:3.

#### Implementation details

The text embedding model based on contrastive learning was implemented using the sentence-transformers library. The pre-trained embedding model selected for fine-tuning is moka-ai/m3e-base, which has been trained on over twenty million Chinese sentence pairs and achieves an optimal balance between encoding performance and inference speed.

For the retrieval-augmented Text-to-SQL generation task, several domestic open-source large language models of varying sizes were selected for comparative analysis. These include DeepSeek-V3-0324 released by DeepSeek, Qwen-2.5-32B-Instruct and Qwen-2.5-7B-Instruct released by Alibaba Cloud, and GLM-4-9B-0414 and GLM-4-32B-0414 released by Zhipu AI. To ensure the reproducibility of our results, the temperature for the LLM was set to 0 to eliminate randomness. The maximum number of self-correction rounds (*N*) was set to 3.

Compared to proprietary models such as ChatGPT, these open-source models offer clear advantages in data security and can be locally deployed to effectively mitigate the risk of data leakage through third-party platforms. Open-source reasoning models are not included in this comparative analysis, as they typically sacrifice some fundamental performance and response speed in pursuit of stronger reasoning capabilities, making them less suitable for the Text-to-SQL task, which requires rapid response.

#### Evaluation metrics.

To directly assess the effectiveness of different example selection strategies, the Jaccard similarity between the SQL queries of selected example questions and those of the target questions is computed. Specifically, database-specific information is first removed from the SQL queries, and then Jaccard similarity is calculated based on the remaining SQL skeletons.

To evaluate the Text-to-SQL generation performance of various LLMs, two metrics are used: exact matching accuracy (EM) and execution accuracy (EX). Exact matching accuracy focuses on the alignment of SQL keywords between the predicted query and the gold-standard query. Execution accuracy, on the other hand, compares the outputs of the predicted and reference SQL queries when executed against the database. Since multiple correct SQL queries may exist for a given question, execution accuracy provides a more precise assessment of model performance.

### Main results

After ten rounds of fine-tuning, the Spearman correlation coefficient for cosine similarity between paired samples in the test set improved significantly, from 0.5230 to 0.9703. As shown in Table 3, example selection strategies exhibited varying performance across 1-shot, 2-shot, and 5-shot settings on the dataset constructed in this study. Notably, increasing the number of examples generally led to lower similarity between the SQL queries of the example questions and the target question, except in the case of random selection. However, the SoftSimMatch strategy consistently outperformed all others, demonstrating a clear advantage in retrieving semantically similar questions within the bibliographic search domain.

**Table 3:** Evaluation of different example selections on BibSQL-test.

Few-shot	Selection	Query similarity
1-shot	Random	0.7994
	Question similarity selection	0.8911
	SoftSimMatch + Question similarity selection	**0.9828**
2-shot	Random	0.7925
	Question similarity selection	0.8818
	SoftSimMatch + Question similarity selection	**0.9825**
5-shot	Random	0.7879
	Question similarity selection	0.8687
	SoftSimMatch + Question similarity selection	**0.9807**

Table 4 compares the Text-to-SQL performance of large language models of different sizes on the BibSQL dataset. Results show that incorporating relevant examples through retrieval-augmented generation (RAG) consistently enhances model performance. As shown in Table 4, the RAG-top1/2/5 methods substantially outperform advanced prompting techniques. In particular, the GLM-4-9B and Qwen-2.5-7B models achieved notable gains, suggesting superior few-shot learning capabilities.

**Table 4:** Comparative evaluation of LLMs on BibSQL-test. Performance is evaluated using various Retrieval-Augmented Generation (RAG) methods, where best results are in bold and second-best are underlined. These methods include using the top 1, 2, or 5 most similar questions (RAG-top1/2/5) and a random dataset sample (RAG-random1) for context.

	Qwen-2.5-7B		GLM-4-9B		GLM-4-32B		Qwen-2.5-32B		DeepSeek-V3	
	EM	EX	EM	EX	EM	EX	EM	EX	EM	EX
w/o RAG	10.4	71.3	7.7	62.0	1.3	62.9	**11.0**	**76.3**	3.5	67.2
RAG-random1	62.2	83.5	70.1	83.0	58.3	82.0	76.3	88.6	**76.5**	**90.6**
RAG-top1	78.2	90.1	84.0	91.8	80.8	92.9	**85.5**	94.1	85.4	**94.3**
RAG-top2	79.8	91.6	87.2	92.4	84.5	93.9	**88.7**	**95.5**	88.2	95.1
RAG-top5	84.9	93.4	91.8	94.5	90.9	96.1	**92.4**	96.3	91.8	**96.6**

Interestingly, even when provided with randomly selected examples (RAG-random1), models performed better than with zero-shot prompting. This indicates that exposure to dataset structure and domain-specific language alone can yield performance benefits, even in the absence of context directly related to the query. However, the marginal benefit diminishes as more context is added: a single retrieved example (RAG-top1) provides a significant boost, while additional examples (RAG-top2, RAG-top5) offer only limited gains, particularly for already high-performing models.

Despite having fewer parameters than DeepSeek-V3, the Qwen-2.5-32B model achieved comparable performance. This can be attributed to DeepSeek-V3’s mixture-of-experts (MoE) architecture, which activates only 37B parameters per inference, similar in scale to Qwen-2.5-32B’s full model. Notably, GLM-4-9B not only outperformed Qwen-2.5-7B but also surpassed its larger counterpart, GLM-4-32B, in exact match accuracy, demonstrating the competitive edge smaller models can have in specific tasks.

### Ablation studies

We conduct an ablation study to assess the impact of PoT approach with RAG-top2 and report the results in Table 5. The PoT approach depends on manually crafted examples, making it costly and impractical to maintain a large-scale example knowledge base in real-world applications. To address this limitation, we manually annotated a small subset of question-SQL pairs from the BibSQL training set, covering all basic question types while deliberately excluding composite ones, and kept the test set unchanged to ensure consistency in evaluation. By using a smaller training set, we more accurately simulate real-world scenarios where exemplar resources are limited.

**Table 5:** Comparative evaluation of LLMs on BibSQL-test with small training set.

	GLM-4-32B		Qwen-2.5-32B		DeepSeek-V3	
	EM	EX	EM	EX	EM	EX
RAG-top2	64.4	83.2	67.7	79.0	**74.8**	**84.4**
PoT + RAG-top2	71.8	85.0	68.3	79.7	**81.4**	**88.1**
Self-correction + PoT + RAG-Top2	73.5	88.6	70.1	83.2	**82.9**	**91.5**

Experimental results reveal a general decline in model performance, particularly in the EM metric. This drop is mainly attributed to the absence of composite questions in the training data, which limits the retrieval of relevant exemplars, even for highly capable RAG module. Among the three models tested, Qwen-2.5-32B exhibited the steepest performance decline and was outperformed by DeepSeek-V3. This suggests that the MoE architecture may possess stronger generalization capabilities when exemplar-based reasoning cannot be directly applied. After incorporating the PoT method, all three models demonstrated notable performance gains, confirming effectiveness of PoT in enhancing model reasoning under limited-resource conditions. Furthermore, the addition of the self-correction mechanism provides another significant boost, particularly to EX score, as it successfully repairs many initially flawed queries, pushing DeepSeek-V3’s EX score past 90%.

To further evaluate the generation performance of each component, we provide a detailed accuracy analysis based on the SQL query structure in Table 6. This analysis breaks down accuracy into four dimensions: *Acc*_*select*_ (correctness of columns in the SELECT clause), *Acc*_*where*_ (correctness of conditions in the WHERE clause), *Acc*_*and*/*or*_ (correctness of logical connectors), and *Acc*_*keywords*_ (correctness of SQL keywords and special operators). The results show that models are highly proficient at identifying the correct columns for selection but face greater challenges with complex WHERE clauses and logical connectors. The PoT and self-correction methods demonstrably improve performance across all dimensions, especially in the more complex *Acc*_*where*_ and *Acc*_*and*/*or*_ categories, validating their ability to enhance logical reasoning and structural accuracy.

**Table 6:** Detailed accuracy of SQL query components on BibSQL-test with small training set.

	$Acc_{select}$	$Acc_{where}$	$Acc_{and/or}$	$Acc_{keywords}$	Average
**GLM-4-32B**					
RAG-top2	95.1	85.3	80.1	88.2	87.2
PoT + RAG-top2	96.0	87.8	83.5	90.1	89.4
Self-correction + PoT + RAG-Top2	96.8	90.2	86.6	91.5	91.3
**Qwen-2.5-32B**					
RAG-top2	92.3	81.1	75.4	84.0	83.2
PoT + RAG-top2	93.5	84.5	79.8	86.7	86.1
Self-correction + PoT + RAG-Top2	94.2	87.0	82.1	88.4	87.9
**DeepSeek-V3**					
RAG-top2	94.8	86.5	82.0	89.3	88.2
PoT + RAG-top2	96.2	89.1	85.4	91.2	90.5
Self-correction + PoT + RAG-Top2	**97.5**	**92.3**	**88.7**	**93.0**	**92.9**

### Error analysis

To better understand the system’s limitations, we conducted a detailed error analysis on the 111 instances where the DeepSeek-V3 + PoT + RAG-top2 configuration failed to produce a correct SQL query on the BibSQL test set. We categorized the types of errors, and Table 7 presents the main error distribution along with representative examples. A detailed analysis of the system’s failures reveals that the primary error types can be grouped into three distinct categories.

**Table 7:** Error analysis of the proposed system on BibSQL-test with small training set.

Error Types	Question, Gold & Prediction	Explanation
Gold Error (30.6%)	**Q:** What are the worthwhile works under the category of psychology? **Gold: ✗** SELECT book FROM book_info WHERE genre = ‘psychology’; **Pred: ✓** SELECT book FROM book_info WHERE genre = ‘psychology’ **ORDER BY rating DESC**;	Judged as incorrect because of the incorrect gold SQL query.
Logic (29.7%)	**Q:** What are the other well - known works of the author of “The Poetic God at Noon”? **Gold: ✓** SELECT **a.author, a.masterpieces FROM author_info a JOIN book_info b ON a.author = b.author WHERE b.book LIKE ‘%The Poetic God at Noon%’**; **Pred: ✗** SELECT *book FROM book_info WHERE author = (SELECT author FROM book_info WHERE book LIKE ‘%The Poetic God at Noon%’) AND book NOT LIKE ‘%The Poetic God at Noon%’*;	The predicted SQL query wrongly assumes that all books are masterpieces.
Inaccuracy (13.5%)	**Q:** What are the tags and ISBN number of “I Want to Waste Time with You”? Could you please elaborate? **Gold: ✓** SELECT **ISBN, tags** FROM book_info WHERE book LIKE ‘%I Want to Waste Time with You%’; **Pred: ✗** SELECT *tags, ISBN, summary* FROM book_info WHERE book = ‘%I Want to Waste Time with You%’;	The selected summary is not asked by the question.
Redundancy (11.7%)	**Q:** I need to know who the author of “A Good Man Is Hard to Find” is and a brief plot introduction of this book. **Gold: ✓** SELECT **summary, author** FROM book_info WHERE book LIKE ‘%A Good Man Is Hard to Find%’; **Pred: ✓** SELECT **b.summary, b.author** FROM book_info b WHERE b.book LIKE ‘%A Good Man Is Hard to Find%’;	Unnecessary alias is added to the syntax structure.
DB Value (10.8%)	**Q:** Which writers were born in Idaho, USA? **Gold: ✓** SELECT author FROM author_info WHERE birthplace LIKE ‘%**Idaho, USA**%’; **Pred: ✗** SELECT author FROM author_info WHERE birthplace LIKE *‘%Idaho%’ AND birthplace LIKE ‘%USA%’; (values written in English)*	Our framework notices ‘Idaho, USA’ is a foreign place and wrongly translates it into English.
Others(3.6%)		

The questions and column values in both the golden and predicted queries were originally written in Chinese and translated into English for presentation, unless otherwise specified.

The first category involves errors in semantic interpretation, namely “Logic” and “Inaccuracy” errors. “Logic” errors occur when the model misinterprets implicit reasoning or relationships within a question, resulting in inaccurate query conditions or structure. This reflects a need for improved handling of complex semantics. “Inaccuracy” errors involve the inclusion of information not requested by the user, which indicates challenges in precise information extraction and controlled generation.

A second class of errors concerns query formulation and data representation, consisting of “Redundancy” and “DB value” errors. “Redundancy” refers to the presence of unnecessary SQL structures, such as excessive aliasing. Although such elements do not affect execution, they can lead to penalties in strict evaluation settings. “DB value” errors arise when the model mismatches values in the database, often due to translation or representation differences. This points to weaknesses in entity alignment.

Finally, a significant portion of failures is attributed to “Gold Error”, a unique type that stems from the evaluation benchmark itself. These errors arise from inconsistencies between the natural language questions and their paired SQL queries in the dataset. In some cases, the model’s output is more semantically appropriate than the gold query but is still judged as incorrect, highlighting limitations in the evaluation standard.

## Conclusions

This study proposes a novel framework that combines RAG and Text-to-SQL techniques to enhance Chinese conversational bibliographic search. By constructing the BibSQL dataset and introducing the SoftSimMatch similarity model, the system significantly improves query understanding and SQL generation accuracy, achieving up to 96.6% execution accuracy. The integration of RAG and PoT prompting proves especially effective under low-resource conditions. This work demonstrates strong generalizability and offers a scalable solution for domain-specific Text-to-SQL applications, laying a foundation for future advancements in intelligent bibliographic retrieval systems.
